# In this month’s *Bulletin*

**DOI:** 10.2471/BLT.14.001014

**Published:** 2014-10-01

In the editorial section, Marie-Renée B-Lajoie et al. (698) explain why Honduras needs to track urban epidemics of injuries and violent deaths more accurately. Iris Borowy (699) argues for WHO’s involvement in drafting sustainable development goals.

Petr Třešňák (702–703) reports on progress towards community-based mental health services in the Czech Republic. Fiona Fleck interviews Daniel Bausch (704–705) on the current Ebola virus disease outbreak in western Africa.

## Burkina Faso

## Is free the right price?

Mira Johri et al. (706–715) model the effects of eliminating user fees for certain health services.

## Finland, Poland & Spain

## Measuring health & happiness

Marta Miret et al. (716–725) analyse findings from a study on ageing.

## Australia, China, Japan, Malaysia, New Zealand, the Philippines, Singapore & Viet Nam

## What works to limit drinking?

Natacha Carragher et al. (726–733) evaluate national alcohol control policies.

## Zambia

## Changes during scale-up

Sujit D Rathod et al. (734–741) track all-cause mortality in communities that have gained access to treatment for HIV.

## Germany

## How feasible is measles elimination?

Anja Takla et al. (742–749) review surveillance data and assess underreporting.

## Bangladesh, Ethiopia, India, Kenya, Malawi, Nepal, the United Republic of Tanzania & Zambia

## Where will they practice?

David M Silvestri et al. (750–759) describe medical and nursing students' reported intentions to work abroad or in rural areas.

## Nepal

## The price of illness

Eiko Saito et al. (760–767) look for causes of catastrophic health spending.

## Global

## Treating streptococcal infections

Rosemary Wyber et al. (768–770) argue for global investment in programmes to prevent rheumatic heart disease.

## Chagas disease in Europe

Yves Jackson et al. (771–772) call for better recognition of, and treatment for, people infected with *T. cruzi.*

